# Efficacy and safety of Lianhua Qingwen granule combined with azithromycin for *mycoplasma* pneumoniae pneumonia in children: a systematic review with meta-analysis and trial sequential analysis

**DOI:** 10.3389/fphar.2024.1374607

**Published:** 2024-06-27

**Authors:** Jiawei Li, Yuqi Ma, Jiawen Qi, Yule Hao, Yiming Wang, Yeke Wu

**Affiliations:** ^1^ School of Basic Medical Sciences, Chengdu University of Traditional Chinese Medicine, Chengdu, China; ^2^ Department of Stomatology, Hospital of Chengdu University of Traditional Chinese Medicine, Chengdu, China

**Keywords:** Lianhua Qingwen granule, azithromycin, *mycoplasma* pneumoniae pneumonia, meta analysis, systematic review, trial sequence analysis

## Abstract

**Background:**

Lianhua Qingwen (LHQW) granule, a botanical drug preparation, is frequently utilized as an adjuvant treatment for *mycoplasma* pneumoniae pneumonia (MPP). Nevertheless, the clinical efficacy and safety of this treatment remain uncertain.

**Purpose:**

This study aims to evaluate the efficacy and safety of LHQW granule combined with azithromycin (AZM) in treating MPP in children.

**Method:**

To identify all randomized controlled trials (RCTs) of LHQW granule plus AZM, a search was conducted in eight Chinese and English databases (CNKI, Wan Fang, VIP, Sinomed, PubMed, Embase, Web of Science, and Cochrane Library) from their inception until 25 December 2023. Meta-regression and subgroup analysis were employed to investigate heterogeneity. Sensitivity analysis and trial sequential analysis (TSA) were conducted to assess the robustness of the findings. Additionally, the Grading of Recommendations Assessment, Development and Evaluation (GRADE) system was utilized to evaluate the quality of evidence.

**Results:**

A total of 15 RCTs involving 1909 participants were included in this study. The meta-analysis results indicated combination therapy of LHQW granule and AZM is significant different from AZM alone in both efficacy and safety, which are specifically observed in the following outcomes: response rate (RR = 1.17, 95% CI: 1.12 to 1.22, *p* < 0.01), antipyretic time (MD = −1.32, 95% CI: −1.66 to −0.98, *p* < 0.01), cough disappearance time (MD = −1.76, 95% CI: −2.47 to −1.05, *p* < 0.01), pulmonary rale disappearance time (MD = −1.54, 95% CI: −2.06 to −1.02, *p* < 0.01), c-reactive protein (CRP) (MD = −5.50, 95% CI: −6.92 to −4.07, *p* < 0.01), procalcitonin (PCT) (MD = −0.31, 95% CI: −0.38 to −0.24, *p* < 0.01), interleukin 6 (IL-6) (MD = −5.97, 95% CI: −7.39 to −4.54, *p*<0.01), tumor necrosis factor α (TNF-α) (MD = −5.74, 95% CI: −7.44 to −4.04, *p* < 0.01), forced vital capacity (FVC) (SMD = 0.48, 95% CI: 0.34 to 0.62, *p* < 0.01), forced expiratory volume in the first second (FEV1) (SMD = 0.55, 95% CI: 0.44 to 0.67, *p* < 0.01), FEV1/FVC (SMD = 0.49, 95% CI: 0.32 to 0.67, *p* < 0.01), CD4^+^ T lymphocyte (CD4^+^) (MD = 4.04, 95% CI: 3.09 to 4.98, *p* < 0.01), CD8^+^ T lymphocyte (CD8^+^) (MD = −3.32, 95% CI: 4.27 to 2.38, *p* < 0.01) and adverse events (RR = 0.65, 95% CI: 0.43 to 0.96, *p* < 0.01).

**Conclusion:**

The combination therapy of LHQW granule and AZM may be a better strategy to treat MPP in children. However, the clinical efficacy and safety of LHQW granule require further validation.

**Systematic Review Registration:**

https://www.crd.york.ac.uk/PROSPERO/.

## Introduction

*Mycoplasma* pneumoniae (MP) is a prokaryotic microbe that lacks a cell wall and is primarily transmitted through air droplets, coughing, and sneezing (Liang et al., 2023). MP is a significant pathogen responsible for *mycoplasma* pneumoniae pneumonia (MPP), a community-acquired infection in children, accounting for up to 40 percent of cases (Mansel et al., 1989; Kumar, 2018; Shah, 2018). Although MPP is commonly observed in school-age children and adolescents and is generally considered benign and self-limiting, it can sometimes progress and result in severe pulmonary complications such as atelectasis, pleural effusion, pulmonary fibrosis, and respiratory distress syndrome. Prompt treatment is crucial to prevent these life-threatening conditions. Macrolides, including erythromycin, clarithromycin, and azithromycin (AZM), are recommended as the first-line therapy for MPP. Among these, AZM has the lowest adverse reaction rate and the longest half-life (Tsai et al., 2021). However, the emergence of macrolide resistance, particularly in Asian regions, has been increasing rapidly in recent years (Lee et al., 2017). Clinical resistance to macrolide antibiotics reduces their efficacy when used alone, leading to delayed treatment and an increased risk of extrapulmonary complications (Wang et al., 2012). Given the global prevalence of MPP and its significant impact on the growth, development, and overall health of affected children, it is imperative to explore more effective clinical treatment strategies.

In China, traditional Chinese medicine (TCM) has been widely used for the treatment of MPP and has shown good tolerability (Sun et al., 2020). Clinical studies have demonstrated that combining TCM with chemical drugs can enhance efficacy and reduce side effects (Yang et al., 2021). Evidence-based guidelines recommend using Lianhua Qingwen (LHQW) granule in combination with conventional treatment for non-severe MMP (Wang et al., 2023). According to the 2020 edition of the Chinese Pharmacopoeia, LHQW granule is a preparation consisting of 13 botanical drugs, including *Forsythia suspensa* (Thunb.) Vahl [Oleaceae; *Forsythiae Fructus*] (Lianqiao, 170 g),* Lonicera japonica* Thunb. [Caprifoliaceae; *Lonicerae Japonicae Flos*] (Jinyinhua, 170 g), *Ephedra sinica* Stapf [Ephedraceae*; Ephedrae Herba*] (Mahuang*,* 57 g), *Prunus armeniaca* L. [Rosaceae; *Armeniacae Semen Amarum*] (Kuxingren, 57 g), *Gypsum Fibrosum* (Shigao, 170 g), I*satis indigotica* Fort. [Cruciferae; *Isatudus Radix*] (Banlangen, 170 g), *Dryopteris crassirhizoma* Nakai [Polypodiaceae; *Dryopteris Crassirhizomatis Rhizoma*] (Mianmaguanzhong, 170 g), *Houttuynia cordata* Thunb. [Saururaceae; *Houttuyniae Herba*] (Yuxingcao, 170 g), *Pogostemon cablin* (Blanco) Benth. [Lamiaceae; *Pogostemonis Herba*] (Guanghuoxiang, 57 g), *Rheum palmatum* L. [Polygonaceae; *Rhei Radix Et Rhizoma*] (Dahuang, 34 g), *Rhodiola crenulata* (Hook. f. et Thoms.) H. Ohba [Crassulaceae; *Rhodiolae Crenulatae Radix Et Rhizoma*] (Hongjingtian, 57 g), *Mentha haplocalyx* Briq. [Lamiaceae; 1-Menthol] (Bohenao, 5 g), *Glycyrrhiza uralensis* Fisch. [Fabaceae; *Glycyrrhizae Radix Et Rhizoma*] (Gancao, 57 g). Pharmacological studies have confirmed the antiviral, antibacterial, anti-inflammatory, and immunomodulatory properties of LHQW (Hu et al., 2022). Furthermore, LHQW has been found to reduce tissue damage caused by inflammatory responses and alleviate pulmonary symptoms (Shen and Yin, 2021; Cao et al., 2022). Compared to traditional decoction, LHQW granule offers the advantages of convenience in carrying, storing, and administration, making it more suitable for children.

Since the outbreak of COVID-19, LHQW has been increasingly used for respiratory infectious diseases in China. However, despite the significant increase in clinical trials combining LHQW granule with AZM for MPP in children in recent years, the efficacy and safety of this combination have not been assessed. To provide evidence for clinical practice, we conducted a systematic review and meta-analysis of 15 latest randomized controlled trials (RCTs) to comprehensively evaluate the efficacy and safety of LHQW granule combined with AZM for MPP in children.

## Methods

This systematic review and meta-analysis followed the Preferred Reporting Items for Systematic Reviews and Meta-Analyses (PRISMA) 2020 guidelines (Page et al., 2021) and the study protocol was registered (CRD42023485960) in the International prospective register of systematic reviews (PROSPERO).

### Search strategy

The literature search for our study included the following subject terms: “Lianhua Qingwen,” “Lianhua Qingwen granule,” “LHQW,” “azithromycin,” “pneumonia, *mycoplasma*,” “*mycoplasma*,” “*mycoplasma* pneumoniae,” “*mycoplasma* pneumoniae pneumonia.” We conducted a comprehensive search of eight databases, including CNKI, Wan Fang, VIP, Sinomed, PubMed, Embase, Web of Science, and Cochrane Library, from inception to 25 December 2023. The search was not restricted by language. Additionally, we conducted a supplementary search of the references of included studies and clinical trial registry to identify any potentially relevant studies. For more details on the search strategy, please refer to the Supplementary Material.

### Inclusion criteria

Participants (P): The participants in this study were children from the ages of 0–14 years. They did not have any other severe diseases such as malignant tumor, severe hepatic and renal insufficiency. There were no restrictions based on gender or race. The clinical diagnostic criteria used in this study were mainly based on the Expert consensus on integrated traditional Chinese and western medicine in the diagnosis and treatment of *Mycoplasma* pneumoniae in children (Liu and Ma, 2017) and Zhu Futang Practice of Pediatrics (Jiang et al., 2015).

Intervention and Control (I and C): The control group received AZM alone, while the experimental group received LHQW granule in addition to AZM. AZM was administered sequentially, orally, and intravenously. Both groups also received other conventional treatments such as defervescence, cough and phlegm reduction, and oxygen inhalation.

Outcome (O): The reported outcomes of the included studies were categorized into three groups: primary outcomes, secondary outcomes, and safety. Primary outcomes focused on clinical efficacy and included measures such as response rate, antipyretic time, cough disappearance time, and pulmonary rale disappearance time. Clinical efficacy was assessed based on physical and laboratory examination results and classified as recovery, remarkable effect, remission, or ineffective: (I) Recovery: Clinical symptoms disappear or almost disappear, laboratory tests return to normal and imaging shows pulmonary reopening. (II) Remarkable effect: Clinical symptoms and laboratory tests improve significantly, reduction in the extent of pulmonary atelectasis by 1/2 or more on imaging. (III) Remission: Clinical symptoms and laboratory tests improve slightly, and imaging shows a reduction in the extent of pulmonary atelectasis by 1/3 or more. (IV) Ineffective: Clinical symptoms and laboratory tests are not improved or even worsened, and the extent of pulmonary atelectasis is reduced by < 1/3 or increased on imaging.The response rate was calculated as the percentage of cases that showed recovery, remarkable effect, or remission out of the total number of cases. Secondary outcomes included various laboratory indicators such as c-reactive protein (CRP), procalcitonin (PCT), Interleukin 6 (IL-6), tumor necrosis factor α (TNF-α), forced vital capacity (FVC), forced expiratory volume in the first second (FEV1), FEV1/FVC, CD4^+^ T lymphocyte (CD4^+^), and CD8^+^ T lymphocyte (CD8^+^). The safety of LHQW granules plus AZM was evaluated by monitoring adverse events.

Study design (S): All the studies included in this analysis were RCTs.

### Exclusion criteria

We excluded studies according to the following criteria: 1) Do not meet the inclusion criteria; 2) Incomplete data; 3) Duplicate studies; 4) Defective study protocol.

### Study selection and data extraction

Two reviewers independently conducted study selection and data extraction. Any disagreements were resolved through consensus or consultation with a third reviewer. Duplicate studies were eliminated, and the reviewers performed preliminary screening by reviewing titles and abstracts. For potentially eligible studies, the full text was thoroughly read for final evaluation. Data extraction included information such as the first author, publication year, sample size, age, course of disease, duration of treatment, diagnostic criteria, intervention measures, and outcome measures.

### Risk of bias assessment

The risk of bias for each included study was independently assessed by two researchers using the Cochrane Risk of Bias (ROB) tool (Higgins et al., 2011). Disagreements were resolved through consensus or discussion with a third researcher. ROB was evaluated based on 7 aspects: random sequence generation, allocation concealment, blinding of participants and personnel, blinding of outcome assessment, completeness of outcome data, selective reporting, and other bias. Each item was rated as low risk, unclear risk, or high risk based on the study characteristics.

### Statistical analysis

The Review Manager 5.4 and Stata 15.0 were used for conducting the meta-analysis. For dichotomous data, the pooled effect size selected was relative risk (RR). For continuous data, mean difference (MD) was chosen as the pooled effect size if the units were uniform; otherwise, standard mean difference (SMD) was selected. Additionally, 95% confidence intervals (CI) were calculated for all pooled results, and a *p*-value <0.05 was considered statistically significant. Heterogeneity among the included studies was assessed using the I2 value. A fixed-effects model was applied when there was low heterogeneity (I^2^ < 50%), while a random-effects model was used for high heterogeneity (I^2^ ≥ 50%). If there was high heterogeneity in the outcomes, meta-regression was performed on multiple covariates to explore potential sources. Subgroup analysis was carried out based on statistically significant covariates (*p* < 0.05) if necessary. Sensitivity analysis was conducted to test the robustness of the results by changing the pooling model and excluding studies one by one. Publication bias was assessed using a funnel plot and Egger’s test when there were at least ten studies for the outcome indicator. The influence of publication bias on pooled results was evaluated using the trim-and-fill method.

### Trial sequential analysis

Random errors and repeated statistical tests in traditional meta-analysis can increase the risk of making type I errors. To address this issue, the Copenhagen Trial Unit has developed Trial Sequential Analysis (TSA) method, which offers several advantages. Firstly, it helps in reducing the risk of type I errors and false-positive findings. Secondly, it estimates the required information size (RIS) for meta-analysis and provides criteria for terminating clinical trials (Kulinskaya and Wood, 2014; Wetterslev et al., 2017). In our study, we conducted trial sequential analysis (TSA) using TSA software (version 0.9.5.10 Beta) based on the results from the meta-analysis. We set the type I errors, relative risk reduction (RRR), and power at 5%, 30%, and 80% respectively.

### Quality of evidence

Grading of Recommendations Assessment, Development and Evaluation (GRADE) system was employed to assess the quality of evidence based on the recommendations (Gonzalez-Padilla and Dahm, 2021). The detailed evaluation items were as follows: risk of bias, inconsistency, indirectness, imprecision and publication bias. The quality of evidence was finally classified into four levels: very low, low, moderate and high.

## Results

### Study selection

**FIGURE 1:**
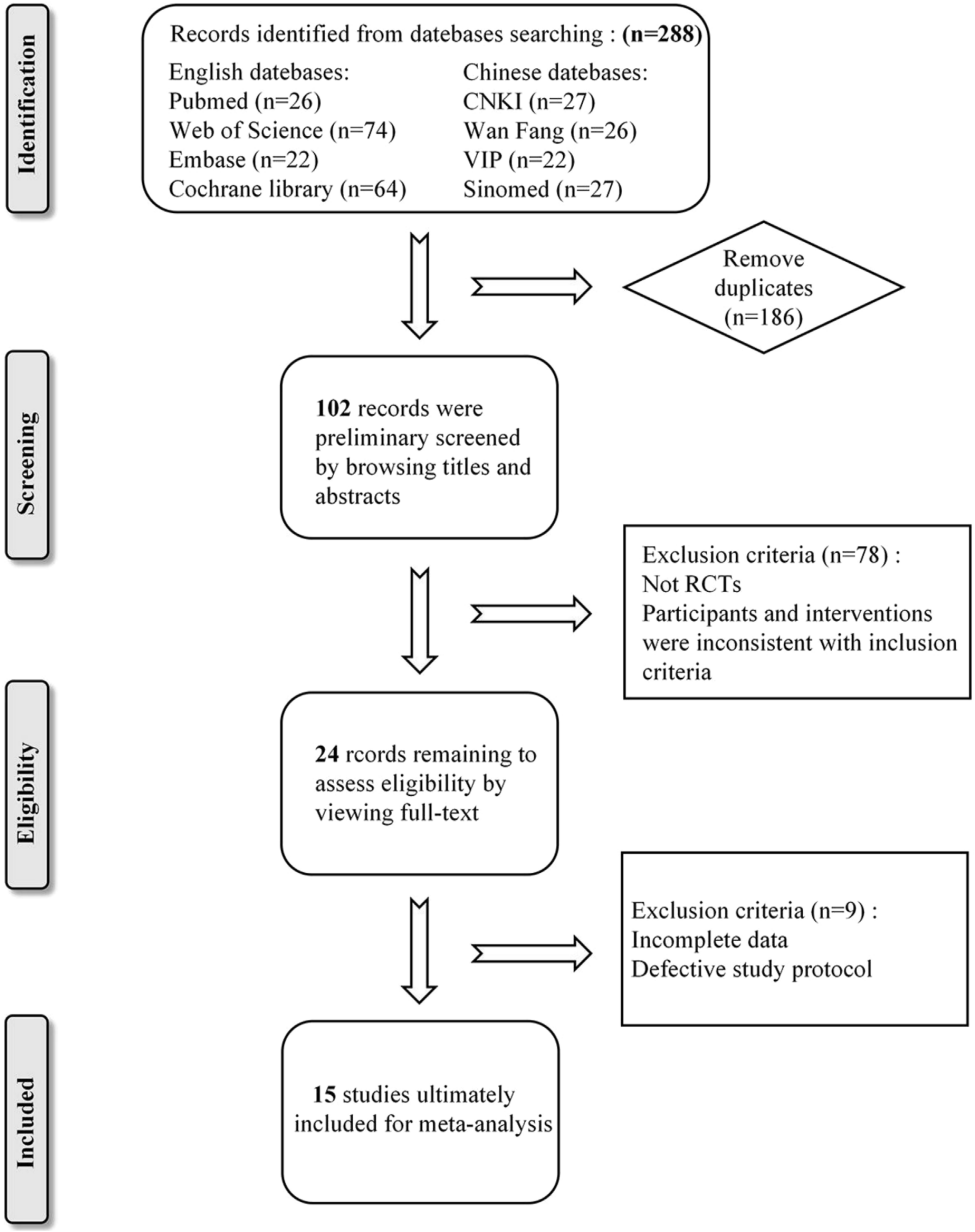
Flow diagram of literature search process.

In accordance with the established search strategy, a total of 288 relevant literatures were retrieved from eight Chinese and English databases. After removing 186 duplicates, the titles and abstracts of 102 studies were read for initial screening. Subsequently, 24 potentially eligible studies were selected for full-text evaluation. Nine studies were excluded due to either lack of pre-treatment data or serious flaws in study design. Ultimately, a total of 15 studies were included in our meta-analysis. Please refer to Figure 1 for a detailed overview of the screening process.

### Characteristics of the included studies

This meta-analysis included a total of 15 (Lin et al., 2019; Wang et al., 2019; Chen et al., 2020; Liu, 2020; Zhu, 2020; Chu, 2021; Zhang and Tuo, 2021; Cheng and Ren, 2022; Ding et al., 2022; Wang et al., 2022; Hu, 2023; Lin et al., 2023; Pi, 2023; Wang, 2023; Zhao et al., 2023) studies conducted in China from 2019 to 2023. All studies were two-arms and single-center RCTs, published in Chinese. The sample sizes ranged from 60 to 260, with a total of 1909 participants, including 957 in the intervention group and 952 in the control group. The participants were children aged 6 months to 14 years, diagnosed with MPP and with a short disease course of no more than a month. In the control group, 11 trials (Lin et al., 2019; Chen et al., 2020; Liu, 2020; Zhu, 2020; Chu, 2021; Zhang and Tuo, 2021; Cheng and Ren, 2022; Wang et al., 2022; Pi, 2023; Wang, 2023; Zhao et al., 2023) used sequential administration of AZM, three trials (Wang et al., 2019; Ding et al., 2022; Lin et al., 2023) used intravenous administration of AZM, and one trial (Hu, 2023) used oral administration of AZM. The intervention group received oral administration of LHQW granule in addition to the standard treatment. Both groups had the same treatment duration of 10–30 days. Further details can be found in Table 1. With reference to the ConPhyMP consensus statement (Heinrich et al., 2022), we have presented summary tables describing the composition, extraction, identification, and product characterization of LHQW granule and how it was reported in the original studies included (Supplementary Tables).

**TABLE 1:** Characteristic of included studies.

Study ID	Sample size (T/C)	Age (years)	Course of disease	Duration (days)	Intervention T	C	Usage of LHQW	Usage of AZM	Outcomes	Diagnostic criteria
Lin et al. (2023)	53/53	T: 7.16 ± 1.02 C: 7.12 ± 1.06	T:16.03 ± 4.18d C:15.50 ± 5.29d	28	AZM + LHQW granule	AZM	3g, tid, po	10 mg/kg·d, qd, ivgtt	①②③④	Ⅳ
Zhao et al. (2023)	100/100	T: 7.62 ± 0.85 C: 7.56 ± 0.82	T: 1.53 ± 0.24w C: 1.47 ± 0.21w	14	AZM + LHQW granule	AZM	Age: ≥8 (3 g), 6–8 (2 g), tid, po	Sequential therapy	①②③④⑤⑥⑧	Ⅰ
Wang et al. (2023)	100/100	T: 7.18 ± 1.00 C: 7.14 ± 1.03	T: 3.10 ± 0.21d C: 3.12 ± 0.23d	30	AZM + LHQW granule	AZM	Age: ≥8 (3 g), 4–7 (2 g), <4 (1 g), tid, po	Sequential therapy	①②③④⑥⑦	Ⅰ
Pi et al. (2023)	41/41	T: 5.90 ± 1.40 C: 5.85 ± 1.32	T: 4.64 ± 1.59d C: 4.58 ± 1.62d	28	AZM + LHQW granule	AZM	3g, tid, po	Sequential therapy	①②③④⑤⑥⑧	Ⅳ
Hu (2023)	43/43	T: 7.49 ± 2.79 C: 7.52 ± 0.81	T: 4.12 ± 0.86d C: 4.15 ± 0.89d	21	AZM + LHQW granule	AZM	Age: ≥10 (6 g), 6–9 (3 g), 3–6 (2 g), <3 (1.5 g), tid, po	10 mg/kg·d, qd, po	②③④⑤⑥	Ⅰ
Wang et al. (2022)	130/130	T: 3.02 ± 1.15 C: 3.04 ± 1.12	T: 2.06 ± 0.31w C: 2.03 ± 0.34w	19	AZM + LHQW granule	AZM	3g, tid, po	Sequential therapy	⑧	Ⅲ
Ding et al. (2022)	30/30	T: 5.87 ± 1.32 C: 5.32 ± 1.01	T: 2.71 ± 0.58d C: 2.38 ± 0.64d	10	AZM + LHQW granule	AZM	Age: ≥7 (6 g), 1–6 (3 g), tid, po	10 mg/kg·d, qd, ivgtt	⑤⑦	Ⅱ
Cheng and Ren (2022)	34/34	T: 5.62 ± 1.50 C: 5.89 ± 1.42	T: 1-28d C: 1-28d	28	AZM + LHQW granule	AZM	Age: ≥8 (3 g), 4–7 (2 g), <4 (1 g), tid, po	Sequential therapy	①②③④⑤⑧	Ⅰ
Zhang and Tuo (2021)	53/50	T: 7.68 ± 2.15 C: 7.41 ± 2.36	T: 3.31 ± 1.06d C: 3.69 ± 1.12d	10	AZM + LHQW granule	AZM	6g, tid, po	Sequential therapy	①⑤⑥⑦⑧	Ⅰ
Chu (2021)	52/52	T: 5.40 ± 1.34 C: 5.63 ± 1.51	T: 2.43 ± 0.51d C: 2.31 ± 0.85d	28	AZM + LHQW granule	AZM	Age: ≥8 (3 g), 4–7 (2 g), <4 (1 g), tid, po	Sequential therapy	①②③④⑤⑥	Ⅰ
Liu (2020)	51/51	T: 7.46 ± 1.25 C: 7.08 ± 1.40	T: 3.47 ± 0.23d C: 3.85 ± 0.10d	21	AZM + LHQW granule	AZM	6g, tid, po	Sequential therapy	①⑤⑧	Ⅳ
Zhu (2020)	58/57	T: 6.36 ± 1.04 C: 6.23 ± 1.08	T: 3.24 ± 0.13d C: 3.21 ± 0.12d	10	AZM + LHQW granule	AZM	Age: ≥10 (6 g), 6–9 (3 g), 3–6 (2 g), <3 (1.5 g), tid, po	Sequential therapy	②③④⑤	Ⅱ
Chen et al. (2020)	79/78	T: 7.81 ± 1.64 C: 7.77 ± 1.53	T: 1.85 ± 0.66w C: 1.89 ± 0.71w	28	AZM + LHQW granule	AZM	Age: ≥8 (3 g), 6- 8 (2 g), tid, po	Sequential therapy	①②③⑤⑥⑦⑧	Ⅱ
Wang et al. (2019)	80/80	T: 5.28 ± 1.53 C: 5.55 ± 1.68	NA	10	AZM + LHQW granule	AZM	6g, tid, po	10 mg/kg·d, qd, ivgtt	①⑤⑥	Ⅱ
Lin et al. (2019)	53/53	T: 7.33 ± 3.42 C: 7.12 ± 2.30	T: 4.23 ± 1.30d C: 4.34 ± 1.41d	19	AZM + LHQW granule	AZM	6g, tid, po	Sequential therapy	①②③④⑤⑥⑦	Ⅰ

Notes: T: treatment group; C: control group; w: week; d: day; LHQW: lianhua qingwen; AZM: azithromycin; ①: response rate; ②: antipyretic time; ③: cough disappearance time; ④: pulmonary rale disappearance time; ⑤: inflammatory indicators (CRP, PCT, IL-6, TNF-α); ⑥: pulmonary function indicators (FVC, FEV1, FEV1/FVC); ⑦: T lymphocytes (CD4^+^, CD8^+^); ⑧: adverse events; Ⅰ: Expert consensus on integrated traditional Chinese and western medicine in the diagnosis and treatment of *Mycoplasma* pneumoniae in children; Ⅱ: Zhu Futang Practice of Pediatrics; Ⅲ: Practical pediatrics; Ⅳ: Conventional diagnostic criteria.

### Risk of bias assessment

**FIGURE 2:**
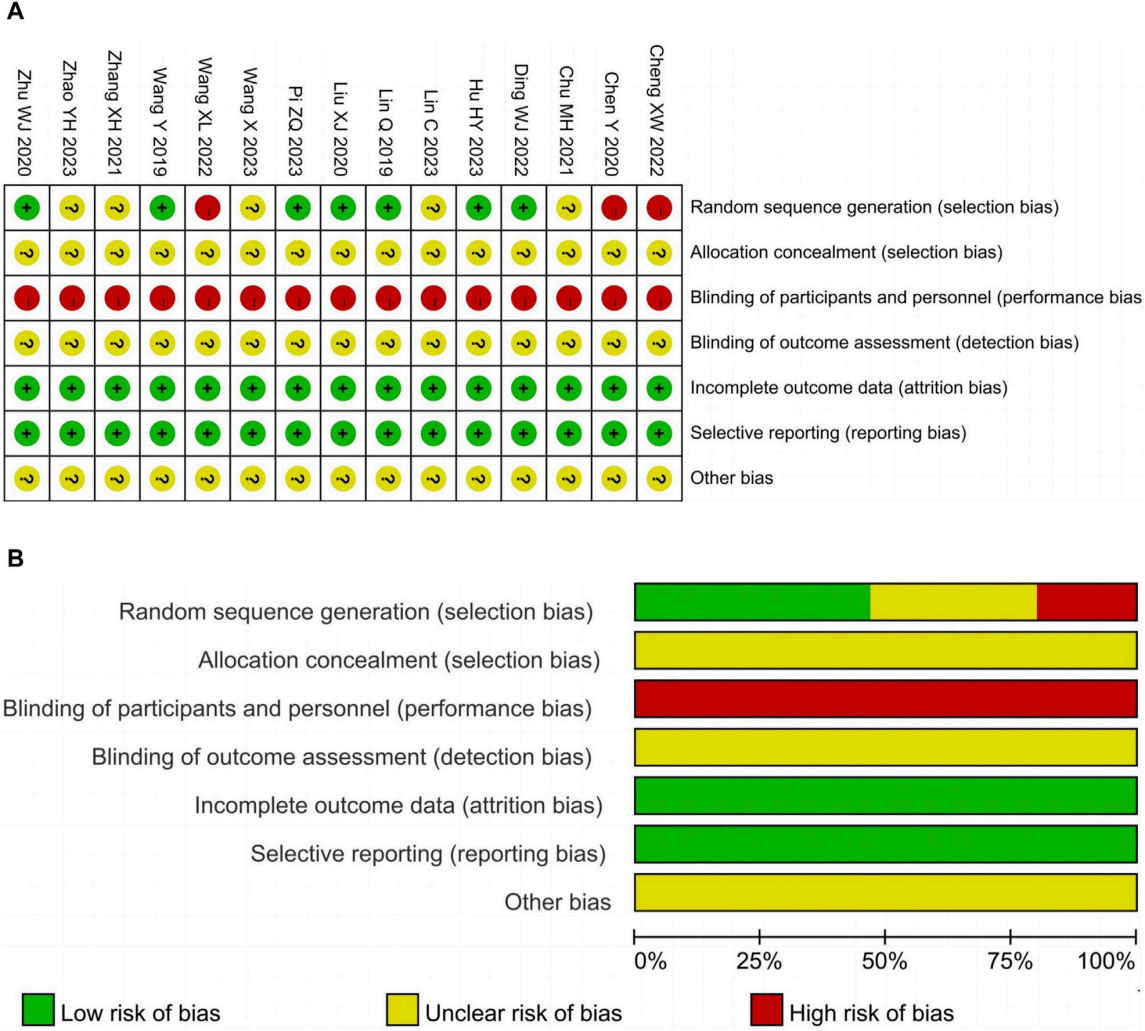
Risk of bias. **(A)** Risk of bias summary. **(B)** Risk of bias graph.

Seven studies (Lin et al., 2019; Wang et al., 2019; Liu, 2020; Zhu, 2020; Ding et al., 2022; Hu, 2023; Pi, 2023) utilized the random number table method for random sequence generation and were classified as low risk. On the other hand, three studies (Chen et al., 2020; Cheng and Ren, 2022; Wang et al., 2022) employed inappropriate randomization methods such as grouping by case number odd or even, resulting in a high risk rating. Furthermore, five studies (Chu, 2021; Chu, 2021; Zhang and Tuo, 2021; Zhang and Tuo, 2021; Lin et al., 2023; Wang, 2023; Zhao et al., 2023) merely mentioned randomization without providing a detailed description of the specific method, leading to an unclear risk assessment. All studies had an unclear allocation concealment and were not conducted in a double-blind manner. In the context of clinical trials, participants can easily determine their group allocation based on the characteristics of TCM such as packaging, smell, and taste. The 15 studies included in the analysis had complete data and did not exhibit selective reporting of outcomes, resulting in a low risk rating. It should be noted that other sources of bias were considered unclear due to insufficient information. The risk of bias assessment results are illustrated in Figure 2.

### Primary outcomes

#### Response rate

**FIGURE 3:**
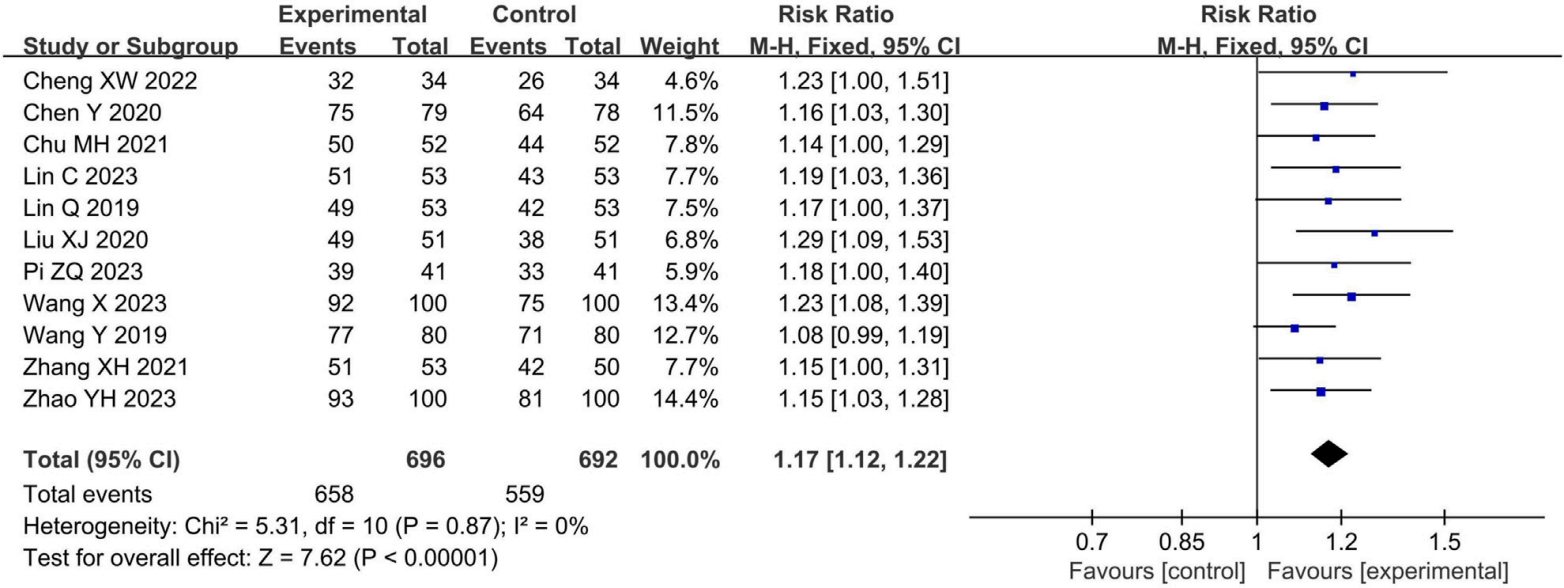
Forest of response rate.

A total of 11 (Lin et al., 2019; Wang et al., 2019; Chen et al., 2020; Liu, 2020; Chu, 2021; Zhang and Tuo, 2021; Cheng and Ren, 2022; Lin et al., 2023; Pi, 2023; Wang, 2023; Zhao et al., 2023) studies involving 1388 participants reported the response rate. The pooled results of fixed-effects model (I^2^ = 0%) showed that LHQW granule combined with AZM significantly improved the response rate compared with AZM alone (RR = 1.17, 95% CI: 1.12 to 1.22, *p* < 0.01; Figure 3).

#### Antipyretic time

**FIGURE 4:**
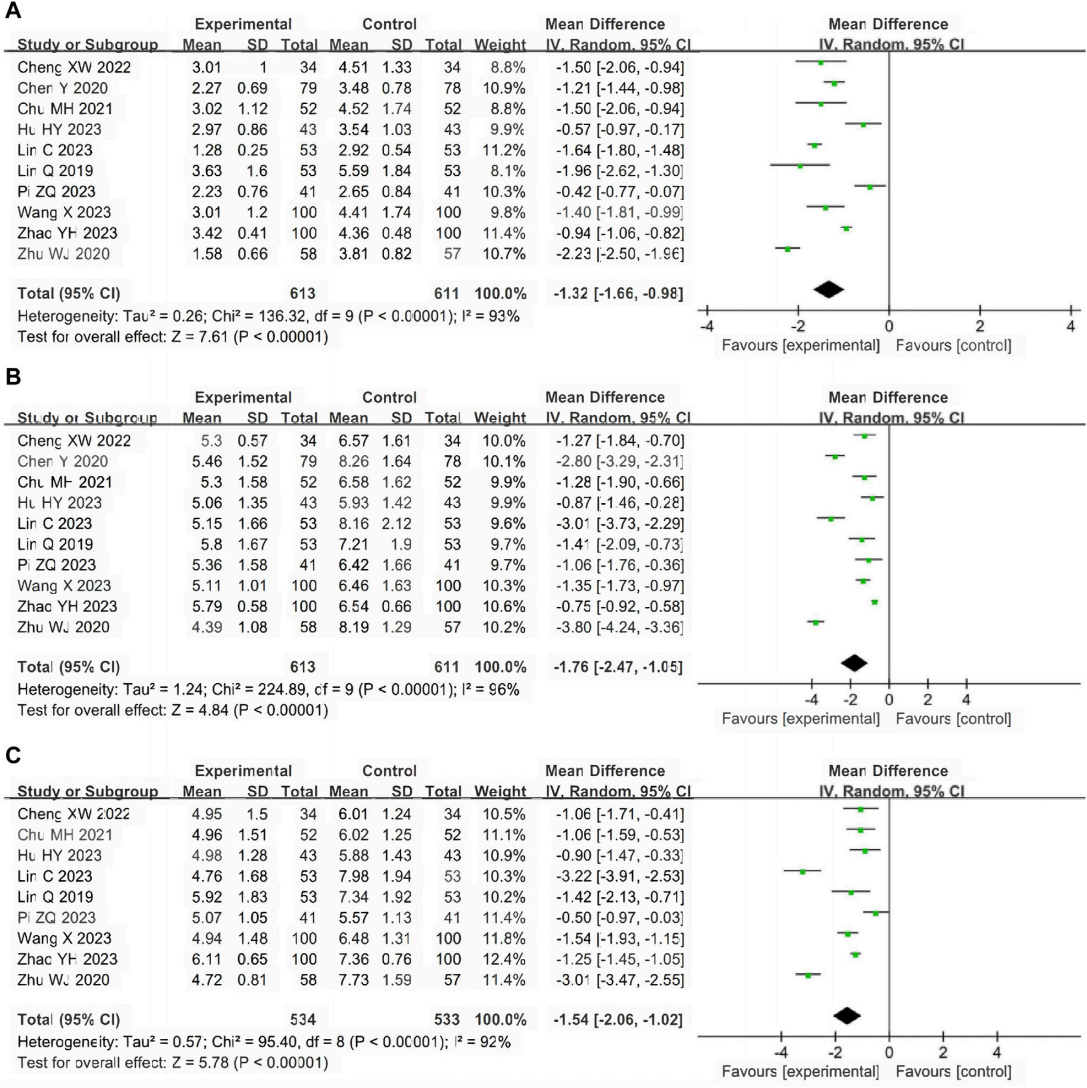
Forest of primary outcomes. **(A)** Antipyretic time. **(B)** Cough disappearance time. **(C)** Pulmonary rale disappearance time.

A total of 10 studies (Lin et al., 2019; Chen et al., 2020; Zhu, 2020; Chu, 2021; Cheng and Ren, 2022; Hu, 2023; Lin et al., 2023; Pi, 2023; Wang, 2023; Zhao et al., 2023) involving 1224 participants reported the antipyretic time. The pooled results of random-effects model (I^2^ = 93%) showed that LHQW granule combined with AZM had better antipyretic effect than AZM alone (MD = −1.32, 95% CI: −1.66 to −0.98, *p* < 0.01; Figure 4A).

#### Cough disappearance time

A total of 10 studies (Lin et al., 2019; Chen et al., 2020; Zhu, 2020; Chu, 2021; Cheng and Ren, 2022; Hu, 2023; Lin et al., 2023; Pi, 2023; Wang, 2023; Zhao et al., 2023) involving 1224 participants reported the cough disappearance time. The pooled results of random-effects model (I^2^ = 96%) showed that LHQW granule combined with AZM had more advantages in relieving cough than AZM alone (MD = −1.76, 95% CI: −2.47 to −1.05, *p* < 0.01; Figure 4B).

#### Pulmonary rale disappearance time

A total of nine studies (Lin et al., 2019; Zhu, 2020; Chu, 2021; Cheng and Ren, 2022; Hu, 2023; Lin et al., 2023; Pi, 2023; Wang, 2023; Zhao et al., 2023) involving 1067 participants reported the pulmonary rale disappearance time. The pooled results of random-effects model (I^2^ = 92%) showed that LHQW granule combined with AZM shortened pulmonary rale disappearance time compared with AZM alone. (MD = −1.54, 95% CI: −2.06 to −1.02, *p* < 0.01; Figure 4C).

### Secondary outcomes

#### Inflammatory indicators

##### CRP

10 studies (Lin et al., 2019; Wang et al., 2019; Chen et al., 2020; Liu, 2020; Zhu, 2020; Chu, 2021; Cheng and Ren, 2022; Ding et al., 2022; Hu, 2023; Pi, 2023) reported CRP levels with 1040 participants. The pooled results of random-effects model (I^2^ = 54%) showed that LHQW granule combined with AZM significantly reduced CRP levels compared with AZM alone (MD = −5.50, 95% CI: −6.92 to −4.07, *p* < 0.01; Supplementary Figure S1A).

##### PCT

Five studies (Lin et al., 2019; Liu, 2020; Chu, 2021; Ding et al., 2022; Zhao et al., 2023) reported PCT levels with 572 participants. The pooled results of fixed-effects model (I^2^ = 0%) showed that LHQW granule combined with AZM significantly reduced PCT levels compared with AZM alone (MD = −0.31, 95% CI: −0.38 to −0.24, *p* < 0.01; Supplementary Figure S1B).

##### IL-6

Nine studies (Lin et al., 2019; Chen et al., 2020; Liu, 2020; Chu, 2021; Zhang and Tuo, 2021; Cheng and Ren, 2022; Ding et al., 2022; Hu, 2023; Pi, 2023) reported IL-6 levels with 982 participants. The pooled results of fixed-effects model (I^2^ = 0%) showed that LHQW granule combined with AZM significantly reduced IL-6 levels compared with AZM alone (MD = −5.97, 95% CI: −7.39 to −4.54, *p* < 0.01; Supplementary Figure S1C).

##### TNF-α

Three studies (Chen et al., 2020; Cheng and Ren, 2022; Hu, 2023) reported TNF-α levels with 311 participants. The pooled results of random-effects model (I^2^ = 88%) showed that LHQW granule combined with AZM significantly reduced TNF-α levels compared with AZM alone (MD = −5.74, 95% CI: −7.44 to −4.04, *p*<0.01; Supplementary Figure S1D).

#### Pulmonary function

##### FEV1

Nine studies (Lin et al., 2019; Wang et al., 2019; Chen et al., 2020; Chu, 2021; Zhang and Tuo, 2021; Hu, 2023; Pi, 2023; Wang, 2023; Zhao et al., 2023) reported FEV1 levels with 1198 participants. The pooled results of fixed-effects model (I^2^ = 0%) showed that LHQW granule combined with AZM significantly increased FEV1 levels compared with AZM alone (SMD = 0.55, 95% CI: 0.44 to 0.67, *p*<0.01; Supplementary Figure S2A).

##### FVC

Six studies (Wang et al., 2019; Chen et al., 2020; Chu, 2021; Zhang and Tuo, 2021; Pi, 2023; Wang, 2023) reported FVC levels with 806 participants. The pooled results of fixed-effects model (I^2^ = 0%) showed that LHQW granule combined with AZM significantly increased FVC levels compared with AZM alone (SMD = 0.48, 95% CI: 0.34 to 0.62, *p*<0.01; Supplementary Figure S2B).

##### FEV1/FVC

Four studies (Lin et al., 2019; Chen et al., 2020; Pi, 2023; Zhao et al., 2023) reported FEV1/FVC levels with 545 participants. The pooled results of fixed-effects model (I^2^ = 0%) showed that LHQW granule combined with AZM significantly increased FEV1/FVC levels compared with AZM alone (SMD = 0.49, 95% CI: 0.32 to 0.67, *p* < 0.01; Supplementary Figure S2C).

#### T lymphocytes

##### CD4^+^

Five studies (Lin et al., 2019; Chen et al., 2020; Zhang and Tuo, 2021; Ding et al., 2022; Wang, 2023) reported CD4^+^ levels with 626 participants. The pooled results of fixed-effects model (I^2^ = 45%) showed that LHQW granule combined with AZM significantly increased CD4^+^ levels compared with AZM alone (MD = 4.04, 95% CI: 3.09 to 4.98, *p* < 0.01; Supplementary Figure S3A).

##### CD8^+^

Five studies (Lin et al., 2019; Chen et al., 2020; Zhang and Tuo, 2021; Ding et al., 2022; Pi, 2023; Zhao et al., 2023) reported CD8^+^ levels with 626 participants. The pooled results of fixed-effects model (I^2^ = 0%) showed that LHQW granule combined with AZM significantly reduced CD8^+^ levels compared with AZM alone (MD = −3.32, 95% CI: 4.27 to 2.38, *p* < 0.01; Supplementary Figure S3B).

#### Safety

**FIGURE 5:**
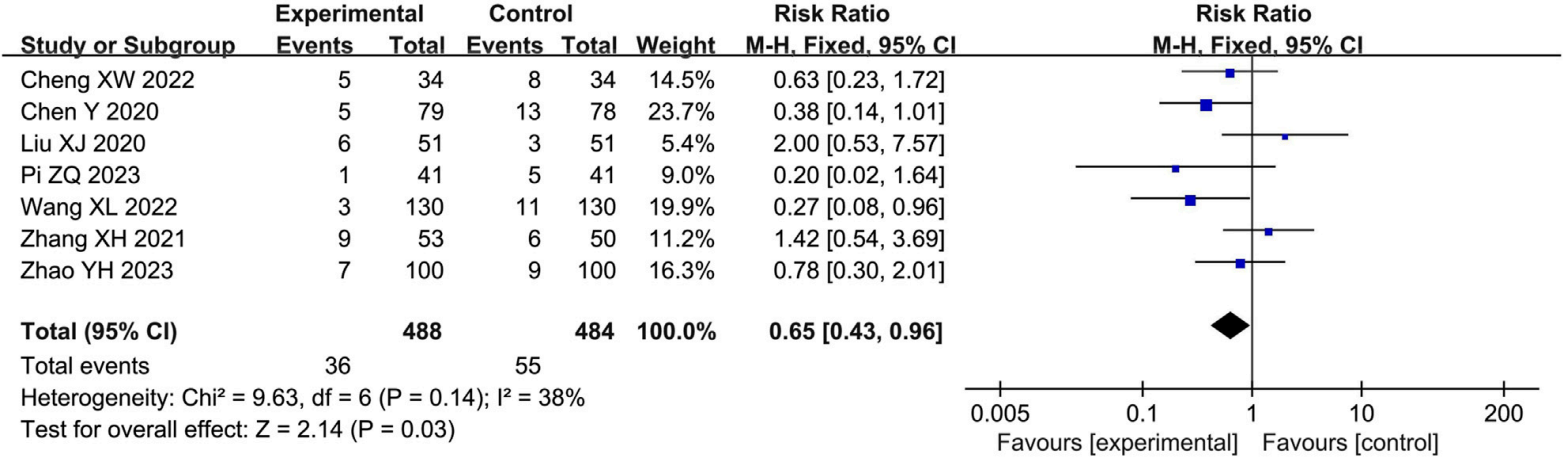
Forest of adverse events.

Seven studies (Chen et al., 2020; Liu, 2020; Zhang and Tuo, 2021; Cheng and Ren, 2022; Wang et al., 2022; Pi, 2023; Zhao et al., 2023) reported adverse events with 972 participants, including rash, headache, dizziness, facial flushing, tachycardia, diarrhea, abdominal pain, nausea and vomiting. The pooled results of fixed-effects model (I^2^ = 38%) showed that LHQW granule combined with AZM significantly reduced the occurrence of adverse events compared with AZM alone (RR = 0.65, 95% CI: 0.43 to 0.96, *p* < 0.01; Figure 5).

### Meta-regression and subgroup analysis

High heterogeneity (I^2^ > 50%) was observed in various factors including antipyretic time, cough disappearance time, pulmonary rale disappearance time, CRP, and TNF-α. To further investigate the potential factors contributing to heterogeneity, we performed meta-regression analysis on the remaining outcomes, excluding TNF-α, due to the limited number of included studies. The meta-regression analysis considered five potential covariates: duration, age, dose of LHQW, course of disease, and sample size. However, the results of the meta-regression analysis indicated that none of these covariates were the source of heterogeneity (Table 2). Consequently, a subgroup analysis of covariates was not conducted.

**TABLE 2:** Results of meta regression analysis.

Outcome	Covariates	Coefficient	Std.err	t	*p* > |t|	95% CI	
Antipyretic time	Duration	0.5025658	0.3893784	1.29	0.233	−0.3953425	1.400474
	Age	0.1466966	0.3936503	0.37	0.719	−0.7610625	1.054456
	Dose of LHQW	−0.3608072	0.4080753	−0.88	0.402	−1.301831	0.5802161
	Course of disease	0.0186424	0.3935761	0.05	0.963	−0.8889457	0.9262305
	Sample size	−0.1714519	0.2417584	−0.71	0.498	−0.7289478	0.386044
Cough disappearance time	Duration	0.3225909	0.7620395	0.42	0.683	−1.434675	2.079857
	Age	0.1932109	0.7202763	0.27	0.975	−1.467749	1.854171
	Dose of LHQW	−0.4181914	0.7583089	−0.55	0.596	−2.166855	1.330472
	Course of disease	−0.2886712	0.7181025	−0.40	0.698	−1.944619	1.367276
	Sample size	−0.2639246	0.4476364	−0.59	0.572	−1.296176	0.7683268
Pulmonary rale disappearance time	Duration	0.5291239	0.6730984	0.79	0.458	−1.062501	2.120749
	Age	−0.2337981	0.662712	−0.35	0.735	−1.800863	1.333267
	Dose of LHQW	−0.3745363	0.6932232	−0.54	0.606	−2.013749	1.264676
	Course of disease	−0.4060672	0.6885221	−0.59	0.574	−2.034163	1.222029
	Sample size	−0.3682318	0.4228167	−0.87	0.413	−1.368034	0.6315708
C-reactive protein	Duration	−0.2411235	1.546641	−0.16	0.880	−3.807685	3.325438
	Age	1.808301	1.358954	1.33	0.220	−1.325453	4.942055
	Dose of LHQW	2.067432	1.433357	1.44	0.187	−1.237895	5.372759
	Course of disease	−3.316321	1.78612	−1.86	0.106	−7.539823	0.9071808
	Sample size	0.0217078	1.020203	0.02	0.984	−2.330885	2.374301

Duration: ≥21 days, <21 days; Age: average age >7 years, average age <7 years; Dose of LHQW: maximum dose (6 g)_tid, po_, maximum dose (3 g)_tid, po_; Course of disease: average course >1 week, average course <1 week; Sample size: number <100, 100 ≤ number ≤150, number >150.

### Publication bias and sensitivity analysis

Egger’s test result (t = 3.71, *p* = 0.005) and the slight asymmetry of the funnel plot suggested the presence of publication bias in the response rate. After confirmation using the trim-and-fill method, five new studies were included. The pooled result remained robust and reliable, as it did not reverse and remained statistically significant (RR = 1.13, 95% CI: 1.10 to 1.17, *p* < 0.01). No publication bias was detected for antipyretic time (t = −0.46, *p* = 0.657), cough disappearance time (t = −0.69, *p* = 0.129), and CRP (t = −1.48, *p* = 0.177). All funnel plots can be seen in Supplementary Figure S4. Sensitivity analysis was conducted for the primary outcomes. The effect sizes of response rate, antipyretic time, cough disappearance time, and pulmonary rale disappearance time were consistent in both the fixed-effects model and random-effects model. Furthermore, when each included study was excluded one by one, the effect size did not change significantly, indicating the stability of the results (Supplementary Figure S5).

### Trial sequential analysis

**FIGURE 6:**
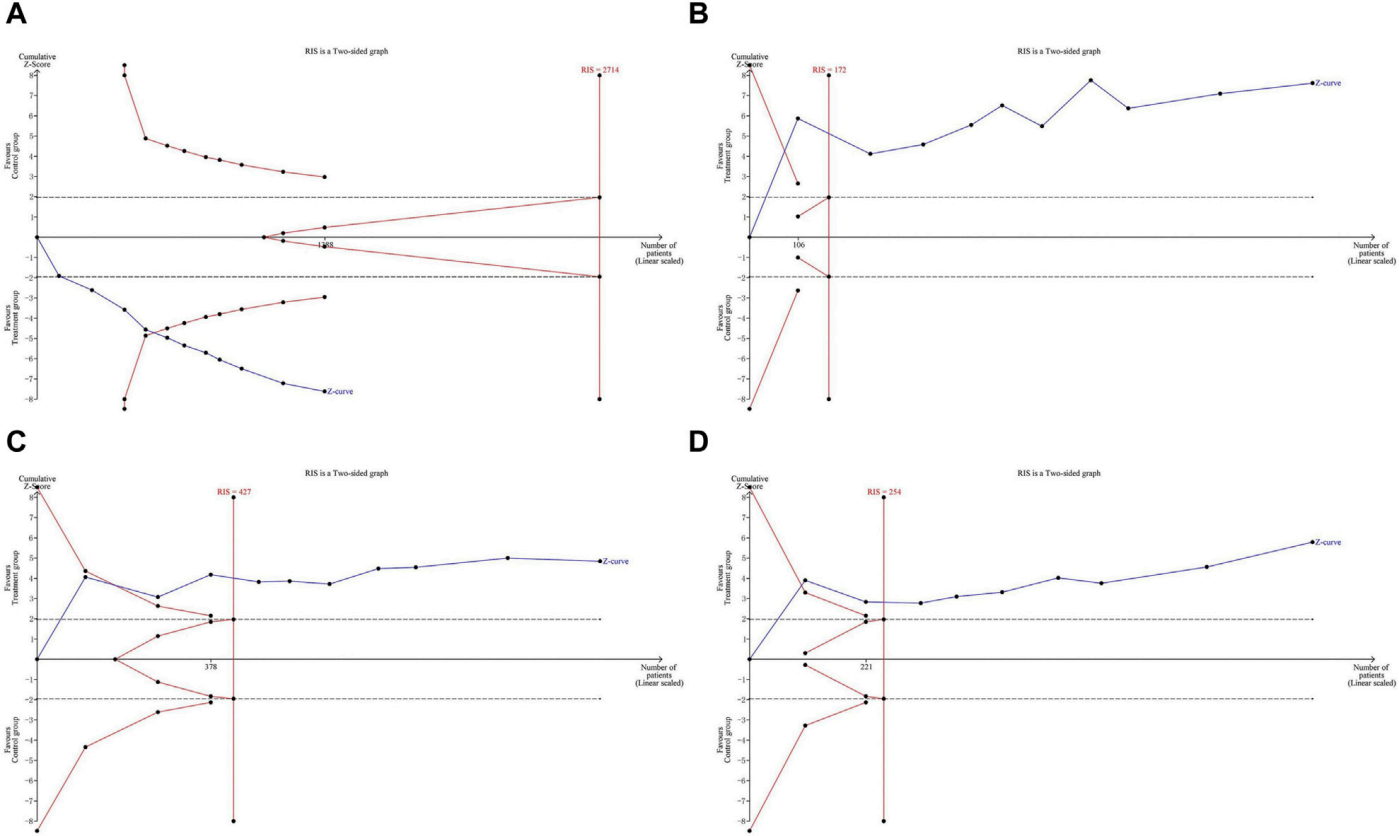
Trial sequential analysis of primary outcomes. **(A)** response rate. **(B)** Antipyretic time. **(C)** Cough disappearance time. **(D)** Pulmonary rale disappearance time.

The meta-analysis results of primary outcomes were used to perform TSA. The Z-curve of response rate crossed both the conventional boundary (dotted lines) and the TSA boundary (red polylines), but it did not reach the RIS (red vertical line). This indicates that the conclusion on response rate is robust, but more studies are needed to further verify it in the future. Regarding antipyretic time, cough disappearance time, and pulmonary rale disappearance time, the Z-curve crossed both the conventional boundary, TSA boundary, and RIS. This confirms that LHQW granule combined with AZM better relieved clinical symptoms of MPP in children compared to AZM alone, without the need for additional studies (Figure 6).

### Quality of evidence

The primary reasons for downgrading the study included the low methodological quality of RCTs, high heterogeneity among the studies, and the presence of publication bias. The results indicated that the quality of evidence for antipyretic time, cough disappearance time, pulmonary rale disappearance time, and TNF-α was assessed as “very low”. The evidence for response rate and CRP was rated as “low,” while the evidence for PCT, IL-6, FEV1, FVC, FEV1/FVC, CD4^+^, CD8^+^, and adverse events was rated as ‘moderate’ (Table 3).

**TABLE 3:** Quality of evidence.

Outcome	Summary of findings				Certainty assessment					
	Study design	Patients (T/C)	Relative effect	Absolute effect	Risk of bias	Inconsistency	Indirectness	Imprecision	Publication bias	Quality
Response rate	RCT (11)	696/692	RR = 1.17 (1.12, 1.22)		Serious^a^	No	No	No	Publication bias strongly suspected	⊕⊕ΟΟ Low
Antipyretic time	RCT (10)	613/611		MD = −1.32 (−1.66, −0.98)	Serious^a^	Very serious^d^	No	No	No Publication bias	⊕ΟΟΟ Very low
Cough disappearance time	RCT (10)	613/611		MD = −1.76 (−2.47, −1.05)	Serious^a^	Very serious^d^	No	No	No Publication bias	⊕ΟΟΟ Very low
Pulmonary rale disappearance time	RCT (9)	534/533		MD = −1.54 (−2.06, −1.02)	Serious^a^	Very serious^d^	No	No	No test	⊕ΟΟΟ Very low
C-reactive protein	RCT (10)	521/519		MD = −5.50 (−6.92, −4.07)	Serious^a^	Serious^c^	No	No	No Publication bias	⊕⊕ΟΟ Low
IL-6	RCT (9)	493/489		MD = −5.97 (−7.39, −4.54)	Serious^a^	No	No	No	No test	⊕⊕⊕Ο Moderate
TNF-α	RCT (3)	156/155		MD = −5.74 (−7.44, −4.04)	Very serious^b^	Very serious^d^	No	No	No test	⊕ΟΟΟ Very low
PCT	RCT (5)	286/286		MD = −0.31 (−0.38, −0.24)	Serious^a^	No	No	No	No test	⊕⊕⊕Ο Moderate
FEV1	RCT (9)	601/597		SMD = 0.55 (0.44, 0,67)	Serious^a^	No	No	No	No test	⊕⊕⊕Ο Moderate
FVC	RCT (6)	405/401		SMD = 0.48 (0.34, 0,62)	Serious^a^	No	No	No	No test	⊕⊕⊕Ο Moderate
FEV1/FVC	RCT (4)	273/272		SMD = 0.49 (0.32, 0,67)	Serious^a^	No	No	No	No test	⊕⊕⊕Ο Moderate
CD4 ^+^	RCT (5)	315/311		MD = 4.04 (3.09, 4.98)	Serious^a^	Not serious	No	No	No test	⊕⊕⊕Ο Moderate
CD8 ^+^	RCT (5)	315/311		MD = −3.32 (−4.27, −2.38)	Serious^a^	No	No	No	No test	⊕⊕⊕Ο Moderate
Adverse events	RCT (7)	488/484	RR = 0.65 (0.43, 0.96)		Serious^a^	Not serious	No	No	No test	⊕⊕⊕Ο Moderate

Notes: T: treatment group C: control group.

^a^ Most of the included studies had a moderate risk of bias.

^b^ Most of the included studies had a high risk of bias.

^c^ *p*-value of the heterogeneity test is too small, and 50% ≤ I2 < 75%.

^d^ *p*-value of the heterogeneity test is too small, and I2 ≥ 75%.

## Discussion

### Summary of evidence

LHQW granule is a contemporary compound TCM preparation with a long history of use in treating respiratory tract infections, developed based on TCM theory and clinical experience. Its metabolites exhibit broad-spectrum antimicrobial activity against various pathogens, including bacteria and viruses. This anti-infective action helps in combating the MP infection directly. Additionally, LHQW contains botanical drugs with potent anti-inflammatory properties. These metabolites work to alleviate the inflammatory response triggered by MP infection, thereby reducing symptoms such as cough, fever, and lung inflammation (Hu et al., 2022). In recent years, there has been extensive research on the active chemical composition of LHQW. High Performance Liquid Chromatography (HPLC) analysis has detected chlorogenic acid, forsythin, amygdalin, and emodin in LHQW preparations. These metabolites are known to have a significant impact on the clinical effectiveness of LHQW (Shao et al., 2022). TNF-α and IL-6 are inflammatory cytokines that are produced during acute inflammation. Numerous studies have confirmed that these cytokines contribute to increased damage caused by infection-induced inflammatory responses, acting through various pathophysiologic pathways. In pharmacological studies, chlorogenic, forsythin, amygdalin, and emodin have been identified as anti-inflammatory metabolites (Dong et al., 2016; Naveed et al., 2018; He et al., 2020; Zhou et al., 2022). In a mouse model of lipopolysaccharide (LPS)-induced acute lung injury (ALI), LHQW preparations were found to reduce lung ultrastructural injury by inhibiting the aggregation of inflammatory cells and reducing levels of various inflammatory cytokines (e.g., TNF-α and IL-6) in serum, thereby protecting lung tissue (Cui et al., 2015a; Cui et al., 2015b). Additionally, *in vitro* experiments have shown that LHQW can reduce the inflammatory response and ameliorate ALI by promoting M2 macrophage infiltration (Li et al., 2023). T lymphocytes play a crucial role in the human immune system. The proportion of CD4^+^ and CD8^+^ T lymphocytes can indicate the immune condition of the body. A decrease in the CD4^+^/CD8^+^ ratio suggests suppressed cellular immune function (Hudson and Wieland, 2023). Research has shown that T lymphocyte dysfunction is often observed in the occurrence and development of MPP, and the severity of MP infection is directly related to the degree of cellular immunosuppression (Luo et al., 2023; Mora and Walczak, 2023). LHQW has been found to have immunomodulatory effects. In mice with influenza virus, LHQW has shown inhibitory effects on the decrease of CD4^+^ and CD4^+^/CD8^+^ levels (Guo et al., 2007). Besides, LHQW preparations can improve pulmonary ventilation and diffusion function by reducing airway inflammation and vascular permeability. Therefore, considering the high clinical resistance of AZM when used alone, combining LHQW granule with AZM is a reasonable strategy for treating MPP in children.

In this study, a total of 15 RCTs involving 1909 participants were included for meta-analysis. The study focused on the combination therapy of LHQW granule and AZM for the treatment of MPP in children. The results indicated that the combination therapy significantly increased the response rate and provided relief for clinical symptoms such as antipyretic time, cough disappearance time, and pulmonary rale disappearance time. It also reduced the levels of serum inflammatory factors including CRP, PCT, IL-6, and TNF-α. Moreover, the combination therapy improved pulmonary function measures such as FEV1, FVC, and FEV1/FVC, as well as immune function indicators CD4^+^ and CD8^+^. However, there was insufficient evidence to suggest that combination therapy could effectively reduce adverse events. Out of the seven studies that reported adverse events, two indicated that combination therapy had lower safety. It is worth noting that both LHQW and AZM have similar adverse reactions, although LHQW only cause slight damage to the gastrointestinal tract and increase the risk of rash (Hu et al., 2022). Furthermore, the quality of evidence for all outcomes ranged from “ery low” to “moderate”. In conclusion, although combination therapy has several advantages, further research is required to validate its efficacy and safety.

### Strengths

Our study has several strengths and potential innovations. Firstly, we believe this is the first systematic review and meta-analysis to comprehensively evaluate the efficacy and safety of LHQW granule plus AZM in the treatment of MPP in children. Secondly, we established strict inclusion and exclusion criteria, specifically selecting only RCTs from the past 5 years to exclude studies of low methodological quality. Thirdly, in contrast to previous meta-analysis studies on MPP, we incorporated additional outcome indicators such as pulmonary function and T lymphocytes to further support the combination therapy of LHQW granule and AZM. We also employed the TSA method and GRADE system to enhance the credibility of our findings. Furthermore, we conducted meta-regression analysis on numerous covariates to explore the source of heterogeneity. Lastly, we exclusively focused on the granular dosage form of LHQW to minimize the heterogeneity of intervention regimes and improve the clinical applicability.

### Limitations

The current study has several limitations that should be acknowledged. Firstly, despite conducting a thorough search of both Chinese and English databases, we were only able to find studies published in Chinese. This may have resulted in a potential bias, as positive results are more likely to be published, while grey literature with negative results may have been missed. Secondly, the majority of the RCTs included in our analysis had limited methodological quality. Specifically, there were issues with the correct use of random allocation methods, unreported allocation concealment, and lack of blinding for both patients and researchers. These methodological deficiencies could introduce selection bias and confounding bias, thereby weakening the strength of the evidence obtained. Thirdly, we observed significant heterogeneity in certain outcomes, such as antipyretic time, cough disappearance time, pulmonary rale disappearance time, and TNF-α. This heterogeneity may be attributed to variations in the diagnostic criteria for MPP and different routes of administration for AZM. In addition, the included trials did not clarify whether the severity of the disease was consistent, which could affect the evaluation of efficacy. Therefore, caution should be exercised when interpreting these findings. Lastly, the lack of adverse events reported in the majority of RCTs and the absence of long-term follow-up data raise concerns regarding the long-term clinical efficacy and safety of LHQW. Further validation is required to deal with these limitations.

### Implications

To address the limitations of previous studies, it is crucial to conduct more high-quality RCTs in the future to assess the efficacy and safety of LHQW in treating MPP in children. We propose the following recommendations: 1) Clinical trials should adhere to the CONSORT 2010 statement (Schulz et al., 2010), and protocols should be registered in advance on a clinical trial platform. 2) The methodological quality of studies should be enhanced. This can be achieved by employing appropriate random allocation methods (such as random number tables or opaque envelopes), ensuring proper allocation concealment, and implementing blinding techniques. 3) Particular attention should be given to monitoring and reporting on the adverse reactions associated with LHQW during the treatment process. Previous research has indicated that certain metabolites of LHQW, such as chlorogenic acid and houttuyfonate, contain allergenic constituents that can cause adverse reactions (Hu et al., 2022). Therefore, it is recommended to adjust the prescription amount of LHQW based on the age and condition of patients in order to minimize the likelihood of adverse reactions.

## Conclusion

Our current study suggests that the combination of LHQW granule and AZM may have better efficacy for MMP in children. However, it is important to note that our findings are limited by low methodological quality and low evidence quality. Therefore, further high-quality studies are needed in the future to validate the clinical efficacy and safety of LHQW.

## Data Availability

The original contributions presented in the study are included in the article/Supplementary Material, further inquiries can be directed to the corresponding author.

## Abbreviations

ALI, Acute lung injury; AZM, Azithromycin; CD4^+^, CD4^+^ T lymphocyte; CD8^+^, CD8^+^ T lymphocyte; CI, confidence intervals; CRP, c-reactive protein; FEV1, forced expiratory volume in the first second; FVC, forced vital capacity; GRADE, the Grading of Recommendations Assessment, Development and Evaluation; HPLC, High Performance Liquid Chromatography; IL-6, Interleukin 6; LHQW, Lianhua Qingwen; LPS, Lipopolysaccharide; MD, mean difference; MPP, *Mycoplasma* pneumoniae pneumonia; MP, *Mycoplasma* pneumoniae; PRISMA, Preferred Reporting Items for Systematic Reviews and Meta-Analyses; PCT, procalcitonin; RCTs, Randomized controlled trials; RIS, required information size; ROB, Risk of Bias; RR, relative risk; RRR, relative risk reduction; SMD, standard mean difference; TCM, traditional Chinese medicine; TNF-α, tumor necrosis factor α; TSA, Trial sequential analysis.
